# Comparison of PNI, HALP, and modified HALP scores in predicting 90-day mortality in elderly patients with acute ischemic stroke

**DOI:** 10.3389/fneur.2026.1765523

**Published:** 2026-03-17

**Authors:** Meliha Fındık, Ramazan Kıyak

**Affiliations:** Department of Emergency, Faculty of Medicine, Balikesir University, Balikesir, Türkiye

**Keywords:** elderly, HALP score, ischemic stroke, modified HALP, Prognostic Nutritional Index

## Abstract

**Introduction:**

Acute ischemic stroke (AIS) remains a major cause of mortality and disability, with older adults disproportionately affected. We aimed to evaluate and compare the prognostic utility of the Prognostic Nutritional Index (PNI), the Hemoglobin–Albumin–Lymphocyte–Platelet (HALP) score, and the modified HALP (mHALP) index for predicting 90-day mortality after AIS in a cohort predominantly composed of elderly patients.

**Methods:**

We conducted a single-center retrospective cohort study including 151 adult patients with radiologically confirmed AIS admitted to the emergency department between January 2021 and December 2024. Demographics, comorbidities, and laboratory parameters obtained within 24 hours of admission were recorded, and baseline stroke severity was assessed using the National Institutes of Health Stroke Scale (NIHSS). PNI, HALP, and mHALP were calculated from routine blood tests. The primary outcome was all-cause 90-day mortality. Associations with mortality were examined using univariate and pre-specified multivariable logistic regression models adjusted for age and NIHSS. Discriminatory performance was assessed using receiver operating characteristic (ROC) curve analysis.

**Results:**

Overall, 26 patients (17.2%) died within 90 days. Non-survivors were older and had significantly lower albumin, hemoglobin, lymphocyte counts, PNI, and HALP values than survivors, and higher NIHSS at presentation. In univariate analyses, PNI, HALP, and mHALP were significantly associated with 90-day mortality. In multivariable models adjusted for age and NIHSS, both PNI and HALP remained independently associated with 90-day mortality, whereas the association for mHALP was attenuated and did not reach conventional statistical significance. ROC analyses indicated fair discrimination for PNI and HALP, modest-to-fair performance for mHALP, and NIHSS performance comparable to a clinical reference.

**Discussion:**

In this AIS cohort, simple immunonutritional indices—particularly PNI and HALP—were independently associated with 90-day mortality and demonstrated fair discriminative ability, supporting their potential role as adjunctive tools for early risk stratification. These findings warrant validation in larger prospective multicenter cohorts and further clarification of the prognostic contribution of mHALP in AIS.

## 1 Introduction

Acute ischemic stroke (AIS) is a leading cause of death and long-term disability worldwide, with older adults bearing a disproportionate share of its fatal and disabling burden. Although established predictors such as age, sex, comorbidities, and baseline stroke severity inform prognosis, they often fail to capture patient-level heterogeneity in risk fully. This limitation has driven interest in complementary, readily obtainable biomarkers that reflect systemic physiology and may refine early risk stratification in the emergency setting (1).

Nutritional and immune status are closely linked to post-stroke recovery, particularly in geriatric patients, in whom malnutrition and systemic inflammation are common and associated with longer hospital stays, complications, and higher mortality (2). The Prognostic Nutritional Index (PNI), calculated from serum albumin and lymphocyte count, has emerged as a pragmatic, low-cost composite of nutritional and immunologic reserve, proposed initially by Onodera et al. (3). Biologically, lower PNI reflects hypoalbuminemia and lymphopenia, which are related to immune dysregulation, heightened inflammation, impaired tissue repair, and increased susceptibility to infection, mechanisms that plausibly worsen short-term outcomes after AIS (2, 4).

Accumulating evidence suggests that low PNI is associated with adverse outcomes in AIS, including in-hospital and short-term mortality and poor functional status at 3 months. For instance, Ustaalioğlu and Umaç (5) reported an association between PNI and in-hospital mortality; Xiang et al. (6) showed that PNI independently predicted 3-month outcomes in patients receiving intravenous thrombolysis; and Aydın and Tatlıparmak (7) identified PNI as a prognostic marker of 30-day mortality in AIS. However, it remains uncertain whether PNI retains its independent prognostic value specifically in elderly patients after accounting for age and comorbidity burden, and how its performance compares with that of other immunonutritional indices in this population.

The hemoglobin–albumin–lymphocyte–platelet (HALP) score is an immunonutritional index that combines measures of erythropoietic capacity, protein reserves, and inflammatory or hematologic activity. In AIS, lower HALP scores have been linked to poorer clinical outcomes and higher rates of post-stroke complications, such as infections, in several studies (8–10). A modified version of this score, the mHALP index, has demonstrated prognostic value in non-stroke conditions such as acute heart failure; however, its usefulness in AIS remains largely speculative, and direct comparative data with PNI and HALP are limited (11).

Several studies have suggested that lower PNI and HALP levels are associated with higher mortality and poorer clinical outcomes, particularly among older patients with acute ischemic stroke. However, data on the prognostic value of the modified HALP (mHALP) score in this setting remain limited. Therefore, this study sought to evaluate and compare the predictive accuracy of the PNI, HALP, and mHALP scores for 90-day mortality in a cohort of patients with AIS, predominantly elderly people.

## 2 Materials and methods

This retrospective cohort study was conducted in the Department of Emergency Medicine at Balıkesir University Faculty of Medicine (Balıkesir, Türkiye) between January 2021 and December 2024. The study was conducted in accordance with the principles of the Declaration of Helsinki and was approved by the Clinical Research Ethics Committee of Balıkesir University Faculty of Medicine (date: 11 March 2025; approval No: 2025/129).

A total of 442 patients diagnosed with acute ischemic stroke (AIS) were screened for eligibility. The inclusion criteria were: (1) age ≥ 18 years; (2) radiologically confirmed AIS on computed tomography (CT) or magnetic resonance imaging (MRI); and (3) availability of complete baseline clinical data, including NIHSS at presentation and laboratory data within the first 24 h of admission. Exclusion criteria were: (1) transient ischemic attack (TIA); (2) hemorrhagic stroke; (3) documented active infection, autoimmune disease, or active malignancy at the time of admission; (4) missing data for any parameter required to calculate PNI, HALP, or mHALP; and (5) prior use of immunosuppressive therapy. After applying these criteria, 151 patients were included in the final analysis.

### 2.1 Data collection and variables

Demographic characteristics (age, sex) and comorbidities (hypertension, diabetes mellitus, atrial fibrillation, coronary artery disease, and history of malignancy) were obtained from electronic medical records. Laboratory parameters, including serum albumin, hemoglobin, lymphocyte count, and platelet count, were recorded from blood samples collected within the first 24 h after admission. Baseline stroke severity was assessed using the National Institutes of Health Stroke Scale (NIHSS) at presentation, as documented in the initial clinical evaluation, and analyzed as a core clinical prognostic covariate. The primary outcome was all-cause mortality at 90 days after the index admission. Ninety-day mortality was defined as death occurring within 90 days; deaths occurring after 90 days were not counted as events for the primary endpoint. As an exploratory secondary endpoint, overall mortality during the available follow-up period was also assessed. Secondary analyses examined the associations between immunonutritional indices (PNI, HALP, and mHALP) and baseline clinico-laboratory characteristics.

### 2.2 Calculation of Immunonutritional indices

All immunonutritional indices were calculated using laboratory values obtained within 24 h of admission.


$$\begin{array}{l} \text{Prognostic Nutritional Index}\;(\mathrm{PNI})=\\ \;\;\;\;\;\;\;\;\;\;\;\;\;\;\;\;\;[10\times \text{serum albumin}\;(g/\mathrm{dL})]\\ +0.005\times \text{total lymphocyte count}\;(\mathrm{per}\;{\mathrm{mm}}^{3}) \end{array}$$

$$\begin{array}{l} \text{Hemoglobin},\text{Albumin},\text{Lymphocyte},\text{and Platelet}\;\\ \left(\text{HALP})\;\text{score}=\text{hemoglobin}\;(g/L)\times \text{albumin}\;(g/L\right)\\ \times \text{lymphocyte count}\;(/L)÷\text{platelet count}\;(/L) \end{array}$$

$$\begin{array}{l} \text{Modified HALP}\;(\text{mHALP})=\log10[\text{hemoglobin}\;(g/\mathrm{dL})\\ \times \text{albumin}\;(g/L)\times \text{lymphocyte count}\;(10^{9}/L)\\ \;\;\;\;\;\;\;\;\;\;\;\;\;\;\;\;\;\;\;\;\;\;\;\times \text{platelet count}\;(10^{9}/L)] \end{array}$$

All formulas were applied in accordance with previously published definitions.

### 2.3 Statistical analysis

Statistical analyses were performed using IBM SPSS Statistics version 25.0 (IBM Corp., Armonk, NY, USA). The distribution of continuous variables was assessed with the Shapiro–Wilk test. Normally distributed variables were expressed as mean ± standard deviation (SD), whereas non-normally distributed variables were presented as median (interquartile range, IQR). Group comparisons were made using the independent-samples *t*-test for normally distributed variables and the Mann–Whitney *U* test for skewed variables. Categorical variables were summarized as counts and percentages and compared using the chi-square test or Fisher’s exact test, as appropriate.

Univariate logistic regression analyses were initially performed to explore the association between candidate predictors and 90-day mortality. To reduce the risk of overfitting, given the limited number of events, multivariable models were prespecified to adjust for age and NIHSS at presentation. To avoid multicollinearity, the composite indices (PNI, HALP, and mHALP) were not included in the model along with their individual laboratory components. Because PNI, HALP, and mHALP are correlated composite measures, they were evaluated in separate multivariable models (age + NIHSS + one index) rather than entered simultaneously.

## 3 Results

**Figure 1:**
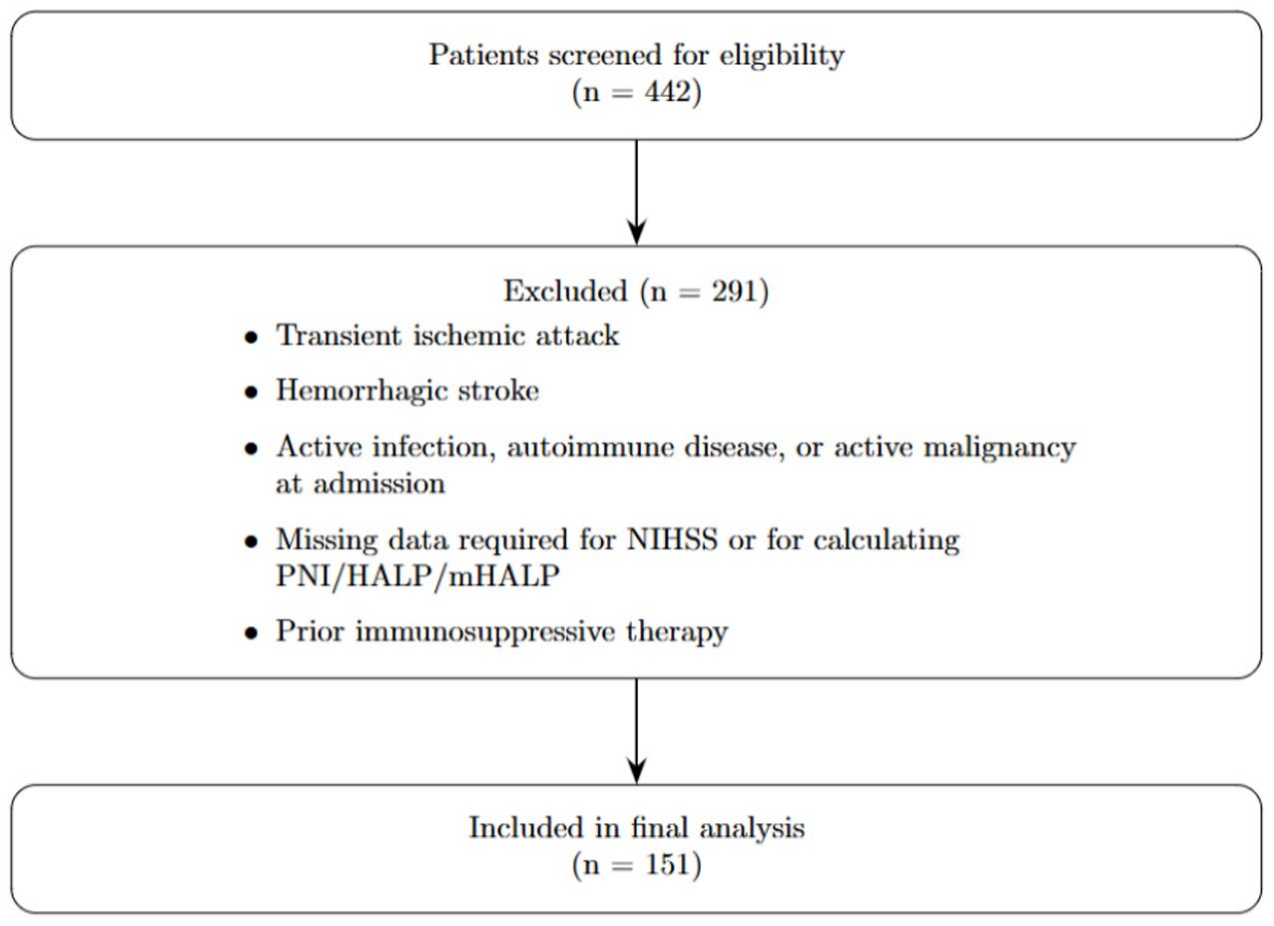
Flow chart of patient selection.

A total of 151 patients were included; 125 (82.8%) were alive at 90 days, and 26 (17.2%) died within 90 days. Patient selection is shown in Figure 1. The mean age was 77.2 ± 9.7 years in the deceased group and 69.8 ± 12.7 years in the survivor group (*p* = 0.002). At presentation, NIHSS was higher in non-survivors than survivors [7.5 (4.0–10.0) vs. 3.0 (2.0–5.0), *p* < 0.001]. At admission, systolic blood pressure was lower in the deceased group (126.3 ± 24.6 vs. 142.1 ± 24.4 mmHg; *p* = 0.009), while diastolic blood pressure was similar between groups (*p* = 0.585). Heart rate was higher in the deceased group (93.0 ± 26.5 vs. 78.9 ± 17.2 bpm; *p* = 0.021). Demographic characteristics, vital signs, and NIHSS are summarized in Table 1.

**Table 1:** Baseline characteristics and vital signs of patients who survived and died within 90 days.

Variable	Survivor (*n* = 125)	Deceased (*n* = 26)	Total (*n* = 151)	*p* value
Female, *n* (%)	62 (49.6%)	18 (69.2%)	80 (53.0%)	0.108
Male, *n* (%)	63 (50.4%)	8 (30.8%)	71 (47.0%)	
Age, years	69.8 ± 12.7	77.2 ± 9.7	71.1 ± 12.5	0.002
NIHSS score	3.0 (2.0–5.0)	7.5 (4.0–10.0)	3.0 (2.0–6.0)	<0.001
Systolic blood pressure, mmHg	142.1 ± 24.4	126.3 ± 24.6	139.2 ± 25.1	0.009
Diastolic blood pressure, mmHg	77.8 ± 13.3	75.9 ± 15.4	77.5 ± 13.6	0.585
Heart rate, bpm	78.9 ± 17.2	93.0 ± 26.5	81.3 ± 19.7	0.021

The distribution of comorbidities is presented in Table 2. Hypertension was the most common comorbidity in the overall cohort. Most comorbid conditions were similarly distributed between survivors and non-survivors; however, a history of malignancy was more frequent among non-survivors (*p* = 0.005).

**Table 2:** Comorbid conditions in patients who survived and died within 90 days.

Comorbidity	Survivor (*n* = 125)	Deceased (*n* = 26)	Total (*n* = 151)	*p* value
Hypertension	75 (60.0%)	13 (50.0%)	88 (58.3%)	0.386
Diabetes mellitus	47 (37.6%)	5 (19.2%)	52 (34.4%)	0.111
Atrial fibrillation	14 (11.2%)	6 (23.1%)	20 (13.2%)	0.117
Coronary artery disease	27 (21.6%)	6 (23.1%)	33 (21.9%)	1.000
Previous cerebrovascular disease	22 (17.6%)	4 (15.4%)	26 (17.2%)	1.000
Hyperlipidemia	4 (3.2%)	1 (3.8%)	5 (3.3%)	1.000
Congestive heart failure	4 (3.2%)	1 (3.8%)	5 (3.3%)	1.000
Chronic kidney disease	1 (0.8%)	0 (0.0%)	1 (0.7%)	1.000
History of malignancy	0 (0.0%)	3 (11.5%)	3 (2.0%)	0.005

Laboratory parameters and immunonutritional indices are summarized in Table 3. Non-survivors had significantly lower hemoglobin and hematocrit levels compared with survivors (11.39 ± 2.84 vs. 12.70 ± 2.02 g/dL, *p* = 0.033; and 34.53 ± 7.99 vs. 38.13 ± 5.60%, *p* = 0.036, respectively). Lymphocyte count was also lower in non-survivors (1.35 ± 0.68 vs. 1.82 ± 0.84 × 10^3^/μL, *p* = 0.003). Serum albumin levels were reduced in the deceased group (33.73 ± 5.75 vs. 37.66 ± 5.49 g/L, *p* = 0.003). Platelet counts were similar between groups (271.81 ± 117.77 vs. 250.26 ± 79.40 × 10^3^/μL, *p* = 0.380).

**Table 3:** Laboratory parameters and immunonutritional indices by 90-day survival status.

Parameter	Survivor (Mean ± SD)	Deceased (Mean ± SD)	Mean difference (95% CI)	*p* value
Hemoglobin, g/dL	12.70 ± 2.02	11.39 ± 2.84	1.31 (0.12 to 2.51)	0.033
Hematocrit, %	38.13 ± 5.60	34.53 ± 7.99	3.60 (0.24 to 6.96)	0.036
Lymphocyte, ×10^3^/μL	1.82 ± 0.84	1.35 ± 0.68	0.48 (0.17 to 0.79)	0.003
Platelet, ×10^3^/μL	250.26 ± 79.40	271.81 ± 117.77	−21.54 (−70.90 to 27.81)	0.380
Albumin, g/L	37.66 ± 5.49	33.73 ± 5.75	3.93 (1.43 to 6.42)	0.003
PNI	46.77 ± 7.41	40.46 ± 6.36	6.31 (3.46 to 9.16)	<0.001
HALP	3.86 ± 2.42	2.16 ± 1.51	1.70 (0.97 to 2.44)	<0.001
mHALP	5.25 ± 0.34	5.01 ± 0.43	0.24 (0.06 to 0.42)	0.011

Consistent with these findings, immunonutritional indices were significantly lower in non-survivors. Mean PNI was 40.46 ± 6.36 in the deceased group and 46.77 ± 7.41 in survivors (*p* < 0.001). HALP values were also reduced in non-survivors (2.16 ± 1.51 vs. 3.86 ± 2.42, *p* < 0.001). The mHALP index was lower in non-survivors compared with survivors (5.01 ± 0.43 vs. 5.25 ± 0.34, *p* = 0.011).

The results of the univariate and multivariable logistic regression analyses for 90-day mortality are shown in Table 4. In univariate analyses, older age [odds ratio (OR) = 1.059, 95% confidence interval (CI) 1.015–1.104, *p* = 0.008], higher NIHSS (OR = 1.226, 95% CI 1.106–1.358, *p* < 0.001), lower PNI (OR = 0.882, 95% CI 0.823–0.945, *p* < 0.001), lower HALP (OR = 0.610, 95% CI 0.449–0.830, *p* = 0.002), and lower mHALP (OR = 0.204, 95% CI 0.066–0.628, *p* = 0.006) were associated with increased risk of 90-day mortality.

**Table 4:** Univariate and prespecified multivariable logistic regression models for 90-day mortality.

Variable	Univariate OR (95% CI)	*p*-value	Multivariable OR (95% CI)	*p*-value
Model 1 (Age + NIHSS + PNI)				
Age (years)	1.059 (1.015–1.104)	0.008	1.016 (0.967–1.069)	0.527
NIHSS (per point)	1.226 (1.106–1.358)	<0.001	1.192 (1.059–1.341)	0.004
PNI (per unit)	0.882 (0.823–0.945)	<0.001	0.890 (0.822–0.963)	0.004
Model 2 (Age + NIHSS + HALP)				
Age (years)	1.059 (1.015–1.104)	0.008	1.017 (0.969–1.068)	0.496
NIHSS (per point)	1.226 (1.106–1.358)	<0.001	1.160 (1.038–1.296)	0.009
HALP (per unit)	0.610 (0.449–0.830)	0.002	0.708 (0.515–0.972)	0.032
Model 3 (Age + NIHSS + mHALP)				
Age (years)	1.059 (1.015–1.104)	0.008	1.027 (0.979–1.077)	0.269
NIHSS (per point)	1.226 (1.106–1.358)	<0.001	1.179 (1.056–1.317)	0.003
mHALP (log-transformed)	0.204 (0.066–0.628)	0.006	0.290 (0.082–1.022)	0.054

In prespecified multivariable models adjusted for age and NIHSS, PNI remained independently associated with 90-day mortality (adjusted OR = 0.890, 95% CI 0.822–0.963, *p* = 0.004), and HALP also remained independently associated (adjusted OR = 0.708, 95% CI 0.515–0.972, *p* = 0.032). mHALP showed an attenuated association after adjustment (adjusted OR = 0.290, 95% CI 0.082–1.022, *p* = 0.054).

**Figure 2:**
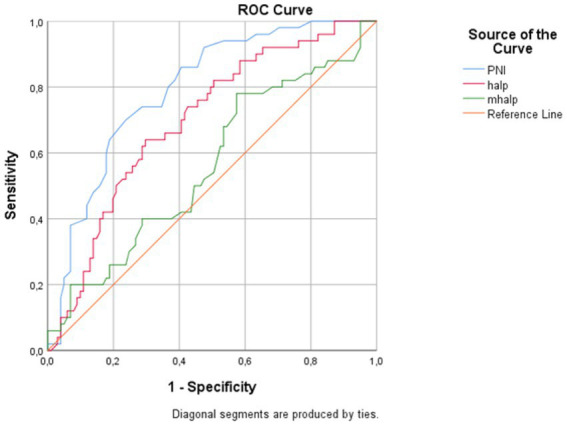
ROC curves for the PNI, HALP, and mHALP in predicting overall mortality during follow-up.

Overall, 50 patients (33.1%) died during the available follow-up period. The diagnostic performance of PNI, HALP, and mHALP for predicting overall mortality during follow-up is presented in Table 5, Figure 2. PNI demonstrated the highest discriminatory ability, with an AUC of 0.793 (95% CI 0.716–0.860, *p* < 0.001); the Youden-optimized cutoff of ≤ 43.00 (*J* = 0.462) yielded 70% sensitivity and 76% specificity. HALP had an AUC of 0.696 (95% CI 0.614–0.783, *p* < 0.001); a cutoff of ≤2.60 (*J* = 0.343) yielded 64% sensitivity and 70% specificity. mHALP showed moderate discriminatory power, with an AUC of 0.678 (95% CI 0.594–0.766, *p* < 0.001); the cutoff of ≤5.351 (*J* = 0.335) provided 86% sensitivity and 48% specificity.

**Table 5:** ROC curve analysis results for PNI, HALP, mHALP, and NIHSS in predicting overall mortality during follow-up.

Score	AUC (95%CI)	Cut-off value	Youden’s *J* index	*p* value	Sensitivity (%)	Specificity (%)
PNI	0.793 (0.716–0.860)	43	0.462	<0.001	70	76
HALP	0.696 (0.614–0.783)	2.6	0.343	<0.001	64	70
mHALP	0.678 (0.594–0.766)	5.351	0.335	<0.001	86	48
NIHSS	0.653 (0.557–0.748)	≥ 4	0.274	0.001	64	63

**Figure 3:**
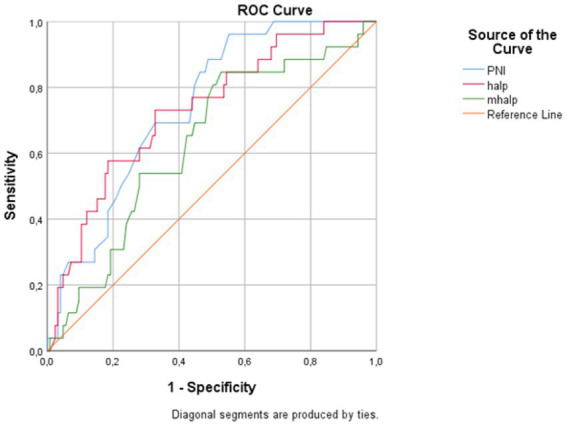
Receiver operating characteristic curves for the PNI, HALP, and mHALP in predicting 90-day mortality.

**Figure 4:**
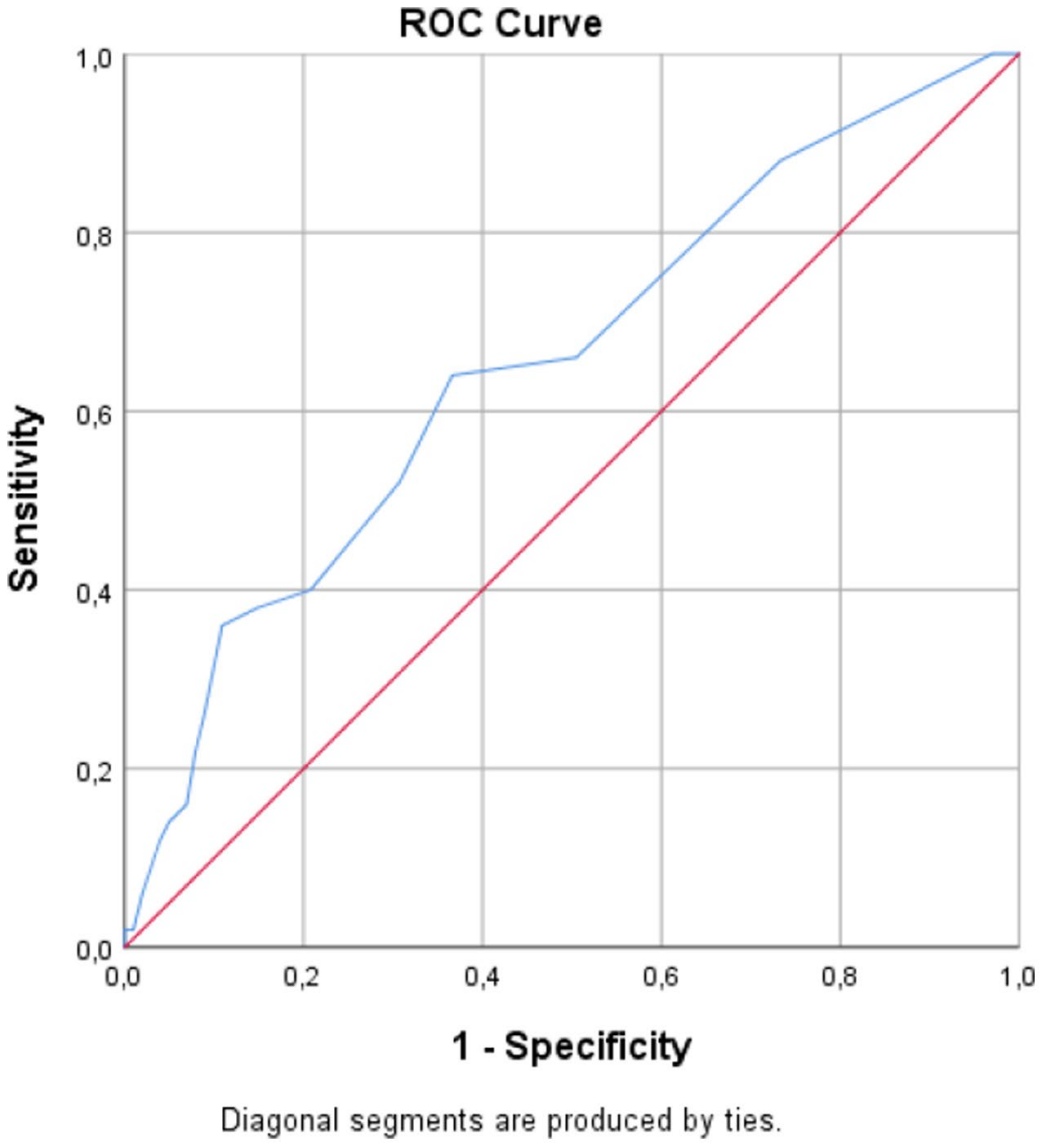
Receiver operating characteristic curve of NIHSS at presentation for predicting overall mortality during follow-up.

**Figure 5:**
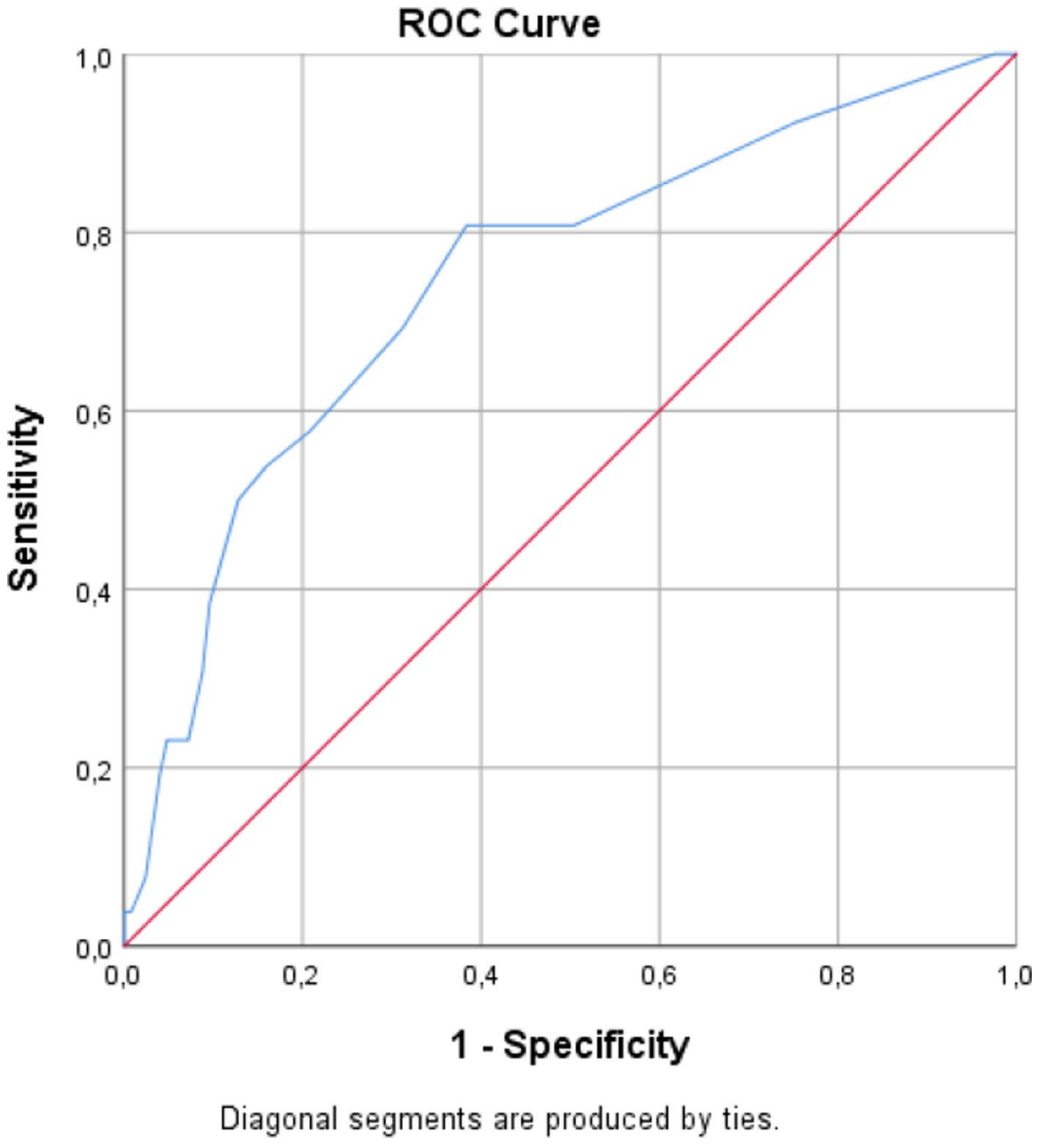
Receiver operating characteristic curve of NIHSS at presentation for predicting 90-day mortality.

The ROC curve analysis for 90-day mortality is summarized in Table 6, Figure 3. PNI had an AUC of 0.742 (95% CI 0.652–0.826, *p* < 0.001); the Youden-optimized cutoff of ≤47.50 (*J* = 0.410) yielded 96% sensitivity and 45% specificity. HALP showed comparable discriminative ability, with an AUC of 0.730 (95% CI 0.622–0.827, p < 0.001); a cutoff of ≤2.41 (*J* = 0.403) yielded 73% sensitivity and 67% specificity. For 90-day mortality, mHALP demonstrated modest-to-fair prognostic value, with an AUC of 0.701 (95% CI 0.601–0.793, *p* < 0.001). The cutoff of ≤5.28 (*J* = 0.461) yielded 88% sensitivity and 58% specificity. For reference, the NIHSS at presentation showed an AUC of 0.746 (95% CI, 0.631–0.855); a cutoff of ≥4 yielded 81% sensitivity and 62% specificity (see Figures 4, 5).

**Table 6:** ROC curve analysis results for PNI, HALP, mHALP, and NIHSS in predicting 90-day mortality.

Score	AUC (95%CI)	Cut-off value	Youden’s *J* index	*p* value	Sensitivity (%)	Specificity (%)
PNI	0.742 (0.651–0.828)	≤47.50	0.410	<0.001	96	45
HALP	0.730 (0.628–0.832)	≤2.41	0.403	<0.001	73	67
mHALP	0.701 (0.594–0.793)	≤5.28	0.461	<0.001	88	58
NIHSS	0.746 (0.628–0.852)	≥4	0.424	<0.001	81	62

## 4 Discussion

Acute ischemic stroke (AIS) prognosis is closely associated with both inflammatory processes and nutritional status. Previous studies have demonstrated that low immunonutritional indices are linked to poor short- and long-term outcomes (5, 7, 10, 12). The Prognostic Nutritional Index (PNI), calculated using serum albumin and lymphocyte counts, was initially proposed in surgical populations (3), and later evaluated in various clinical contexts, including cerebrovascular disease. Similarly, the HALP score reflects oxygen-carrying capacity, protein reserves, and inflammatory or hematologic responses. Originally developed in oncology (13). HALP has been associated with outcomes in stroke as well (10). More recently, mHALP has been introduced to improve discriminative ability by rescaling component weights. However, the lack of a standardized formula has led to methodological heterogeneity and highlighted the need for external validation (11). Beyond cerebrovascular disease, immunonutritional indices have shown prognostic relevance in other cardiovascular conditions, such as HALP predicting long-term mortality in non-valvular atrial fibrillation (14) and NLR being associated with adverse outcomes in heart failure (15).

Emerging evidence supports the prognostic role of immunonutritional scores in cerebrovascular disease. Shestopalov et al. (16) reported that malnutrition risk was significantly associated with mortality and poor functional outcomes in neurocritical care cohorts, including stroke patients. Similarly, Ramesh et al. (17) demonstrated that HALP was strongly correlated with mortality and functional disability in AIS. These findings suggest that immunonutritional indices capture both inflammatory and hematologic activity and underlying nutritional reserves, thereby offering additional prognostic information. In line with these observations, our results confirm the prognostic relevance of PNI and HALP in elderly patients with AIS.

In the present study, both PNI and HALP were significantly lower in non-survivors and were associated with 90-day mortality in univariate analyses. PNI remained an independent predictor of 90-day mortality after adjustment for age and significant comorbidities, whereas HALP and mHALP did not retain statistical significance in the multivariable model. Although mHALP showed a statistically significant association with 90-day mortality in ROC analysis, its AUC value (0.63) indicated only modest discriminatory capacity. Taken together, these findings suggest that PNI and HALP may provide valuable prognostic information in elderly AIS patients, while the clinical utility of mHALP in this setting appears limited (18, 19).

These results are broadly consistent with those of Wang et al. (20), who observed an inverse association between PNI and 90-day mortality in a large prospective AIS cohort. In other clinical fields, recalibration and optimization of HALP and related indices using advanced methods, including machine learning, have been reported. Monarchi et al. (18) enhanced the predictive performance of HALP in reconstructive surgery by applying machine–learning–based modifications, achieving AUC values above 0.90. In contrast, Lin et al. (19) showed that mHALP outperformed traditional HALP and qSOFA in sepsis cohorts. In oncology, low HALP has been associated with worse overall and progression-free survival in non-small cell lung cancer, particularly among elderly patients with higher tumor burden (21, 22). Although these studies were conducted in different populations, they collectively support the concept that composite immunonutritional indices are clinically meaningful markers of adverse outcomes, especially in older and comorbid patients.

The performance differences between HALP and mHALP in this study may partly be explained by their mathematical structures and the biological roles of platelets. In the original HALP score, platelet count is in the denominator, so higher platelet counts, which may reflect increased thrombo-inflammatory activity, are associated with lower HALP values and a worse prognosis. By contrast, in mHALP, platelet count is a multiplicative factor, potentially altering its biological interpretation in conditions where thrombo-inflammatory mechanisms play a central role, such as AIS or acute coronary syndromes. This may contribute to mHALP’s weaker and less consistent prognostic performance in this cohort. In addition, PNI is based solely on albumin and lymphocyte counts, two parameters directly linked to nutritional and immunological status, whereas HALP and mHALP also incorporate hemoglobin and platelet counts, which can be influenced by comorbidities and hematologic conditions not directly related to stroke outcomes (6, 8, 23). Such variability may attenuate the robustness of HALP and mHALP in elderly AIS populations with multiple comorbidities and hematologic variability.

From a clinical perspective, the present findings suggest that PNI and, to a lesser extent, HALP could be considered adjunctive tools for early risk stratification in elderly patients with AIS. These indices are inexpensive, rely on routinely available laboratory parameters, and can be calculated shortly after admission, which makes them attractive for use in emergency and acute stroke settings. However, given the modest discriminatory performance observed and the lack of external validation, they should not be regarded as replacements for established clinical prognostic tools. Instead, PNI and HALP may complement existing risk stratification strategies by providing additional information on the combined impact of nutritional and inflammatory status. Further prospective multicenter studies are needed to validate these findings, clarify the role of mHALP in cerebrovascular disease, and determine whether recalibrated or machine-learning–derived versions of HALP and mHALP, when integrated with imaging and inflammatory biomarkers, can yield more accurate and clinically actionable prognostic models.

This study has several limitations. First, its retrospective, single-center design limits causal inference and increases the risk of selection bias and unmeasured confounding. Second, although baseline stroke severity was incorporated using the National Institutes of Health Stroke Scale, infarct characteristics could not be included as standardized covariates. While acute ischemic stroke was radiologically confirmed and radiology reports were available, imaging was interpreted and reported by different radiologists in routine clinical practice without a uniform structured template; therefore, consistent extraction of key prognostic imaging features such as vascular territory, infarct volume or extent, ASPECTS, large-vessel occlusion status, hemorrhagic transformation, and edema or mass effect would require standardized neuroradiologic re-adjudication, which was not feasible in this study. Third, data on acute reperfusion therapies, including intravenous thrombolysis and mechanical thrombectomy, were not available in a structured format for modeling; moreover, our institution is not a comprehensive stroke center and many patients presented beyond guideline-recommended therapeutic windows, likely limiting treatment eligibility and introducing pathway-related heterogeneity. Fourth, the sample size—particularly the number of deaths within 90 days—was modest, limiting statistical power and potentially affecting model stability despite conservative modeling and evaluation of each immunonutritional index in separate adjusted models. Fifth, we assessed all-cause 90-day mortality only; functional outcomes such as the modified Rankin Scale could not be evaluated, limiting conclusions regarding post-stroke disability. Finally, external validation was not performed. Accordingly, these findings should be considered hypothesis-generating and require confirmation in larger, prospective, multicenter cohorts—ideally including comprehensive stroke centers—with standardized imaging phenotyping, detailed reperfusion treatment data, and concurrent evaluation of disability outcomes, including 90-day modified Rankin Scale.

## 5 Conclusion

This study demonstrates that lower PNI and HALP scores are associated with increased 90-day mortality in elderly patients with acute ischemic stroke (AIS), and that PNI and HALP remain independently associated with 90-day mortality after adjustment for age and baseline NIHSS, whereas mHALP shows an attenuated (borderline) association and requires further evaluation. Immunonutritional indices derived from routine laboratory tests may therefore serve as adjunctive tools for early risk stratification in elderly patients with AIS. Further prospective, multicenter studies are warranted to validate these findings, incorporate imaging and reperfusion variables, and determine whether refined or recalibrated score formulations can meaningfully improve prognostic accuracy in this population.

## Data Availability

The raw data supporting the conclusions of this article will be made available by the authors, without undue reservation.

## References

[1] Powers WJ Rabinstein AA Ackerson T Adeoye OM Bambakidis NC Becker K . Guidelines for the early management of patients with acute ischemic stroke: 2019 update to the 2018 guidelines for the early management of acute ischemic stroke, a guideline for healthcare professionals from the American Heart Association/American Stroke Association. Stroke. (2019) 50:E344–418. doi: 10.1161/STR.0000000000000211, 31662037

[2] Nergiz S Ozturk U. The effect of prognostic nutritional index on infection in acute ischemic stroke patients. Medicina (Kaunas). (2023) 59:679. doi: 10.3390/medicina59040679, 37109637 PMC10143634

[3] Onodera T Goseki N Kosaki G. Prognostic nutritional index in gastrointestinal surgery of malnourished cancer patients. Nihon Geka Gakkai Zasshi. (1984) 85:1001–5. 6438478

[4] He J Yin H Xia Y Wu JZ Liang JH Zhu HY . Prognostic nutritional index, a novel biomarker which predicts worse prognosis in diffuse large B cell lymphoma. Leuk Res. (2021) 110:106664. doi: 10.1016/J.LEUKRES.2021.106664 34271293

[5] Ustaalioğlu İ Umaç GA. The role of the prognostic nutritional index in predicting mortality in stroke patients. Rev Assoc Med Bras. (2024) 70:e20240714. doi: 10.1590/1806-9282.20240714, 39292077 PMC11404984

[6] Xiang W Chen X Ye W Li J Zhang X Xie D. Prognostic nutritional index for predicting 3-month outcomes in ischemic stroke patients undergoing thrombolysis. Front Neurol. (2020) 11:599. doi: 10.3389/fneur.2020.00599, 32670192 PMC7333017

[7] Aydın ÖF Tatlıparmak AC. Prognostic nutritional index as a predictor of mortality in acute ischemic stroke. Clin Neurol Neurosurg. (2025) 249:108750. doi: 10.1016/J.CLINEURO.2025.108750, 39847887

[8] Ozturk U Nergiz S Ozturk O. The association between HALP score and infection in acute ischemic stroke patients. J Stroke Cerebrovasc Dis. (2024) 33:107929. doi: 10.1016/J.JSTROKECEREBROVASDIS.2024.107929, 39159902

[9] Zhu X Zhang Y Wang A Zhang X Yu G Xiang S . Association between HALP (hemoglobin, albumin, lymphocyte, and platelet) score and poor outcomes in acute ischemic stroke patients with type 2 diabetes mellitus: a study from the third China National Stroke Registry. Front Neurol. (2024) 15:1461188. doi: 10.3389/fneur.2024.1461188, 39839876 PMC11746045

[10] Tian M Li Y Wang X Tian X Pei LL Wang X . The hemoglobin, albumin, lymphocyte, and platelet (HALP) score is associated with poor outcome of acute ischemic stroke. Front Neurol. (2021) 11:610318. doi: 10.3389/fneur.2020.610318, 33510706 PMC7835486

[11] Kocaoglu S Alatli T. The efficiency of the HALP score and the modified HALP score in predicting mortality in patients with acute heart failure presenting to the emergency department. J Coll Physicians Surg Pak. (2022) 32:706–11. doi: 10.29271/jcpsp.2022.06.706 35686400

[12] Bolayır A. Can the prognostic nutritional index be used as an indicator of mortality in patients with acute ischemic stroke? Cumhur Med J. (2020) 42:484–90. doi: 10.7197/cmj.834780

[13] Chen X-L Xue L Wang W Chen H-N Zhang W-H Liu K . Prognostic significance of the combination of preoperative hemoglobin, albumin, lymphocyte and platelet in patients with gastric carcinoma: a retrospective cohort study. Oncotarget. (2015) 6:41370–82. doi: 10.18632/oncotarget.5629, 26497995 PMC4747412

[14] Söner S Güzel T Aktan A Kılıç R Söner HT Demir M . Prognostic value of hemoglobin, albumin, lymphocyte, platelet (HALP) scores in patients with non-valvular atrial fibrillation: insights from the AFTER-2 study. BMC Cardiovasc Disord. (2025) 25:528. doi: 10.1186/s12872-025-04993-1, 40684097 PMC12275269

[15] Vakhshoori M Nemati S Sabouhi S Yavari B Shakarami M Bondariyan N . Neutrophil to lymphocyte ratio (NLR) prognostic effects on heart failure; a systematic review and meta-analysis. BMC Cardiovasc Disord. (2023) 23:555. doi: 10.1186/s12872-023-03572-6, 37957565 PMC10644447

[16] Shestopalov AE Yakovleva AV Yadgarov MY Sergeev IV Kuzovlev AN. Prevalence and impact of malnutrition risk on outcomes in critically ill patients with traumatic brain injury and stroke: a retrospective cohort study using electronic health records. Nutrients. (2024) 16:2396. doi: 10.3390/nu16152396, 39125277 PMC11314111

[17] Ramesh NB Rangan AS Thirugnanam G Kumar Kalappan M. Correlation of hemoglobin, albumin, lymphocyte, and platelets (HALP) score with outcome of acute ischemic stroke. Cureus. (2024) 16:e67979. doi: 10.7759/cureus.67979 39347246 PMC11427763

[18] Monarchi G Committeri U Gilli M Salzano G Troise S Consorti G . Evolution and optimization of the HALP formula for predicting free flap failure: a progressive analysis of predictive accuracy. Surgeries. (2025) 6:44. doi: 10.3390/surgeries6020044

[19] Lin L Huang H Wu M Chen F Li C. The modified HALP score is associated with short-term mortality in critically ill patients with sepsis—a cohort study. J Infect Dev Ctries. (2025) 19:924–33. doi: 10.3855/jidc.20755, 40608725

[20] Wang J Cao X Zeng S Zhou L Huang J Han Y . Nonlinear dose–response relationship between prognostic nutritional index and short-term outcome in acute ischemic stroke: a prospective cohort study. Front Nutr. (2025) 12:1529146. doi: 10.3389/fnut.2025.1529146, 40129670 PMC11930808

[21] Li L Zhang H Yang Q Chen B. The effect of prognostic nutritional indices on stroke hospitalization outcomes. Clin Neurol Neurosurg. (2024) 247:108642. doi: 10.1016/J.CLINEURO.2024.108642 39561581

[22] Shen XB Zhang YX Wang W Pan YY. The hemoglobin, albumin, lymphocyte, and platelet (HALP) score in patients with small cell lung cancer before first-line treatment with etoposide and progression-free survival. Med Sci Monit. (2019) 25:5630–9. doi: 10.12659/MSM.917968, 31356586 PMC6685331

[23] Jiang TT Zhu XY Yin YW Liu HJ Zhang GY. The prognostic significance of malnutrition in older adult patients with acute ischemic stroke. Front Nutr. (2025) 12:1529754. doi: 10.3389/fnut.2025.1529754, 39957766 PMC11825317

